# Expression of Toll-Like Receptors on Breast Tumors: Taking a Toll on Tumor Microenvironment

**DOI:** 10.1155/2012/716564

**Published:** 2011-11-09

**Authors:** Debika Bhattacharya, Nabiha Yusuf

**Affiliations:** ^1^Department of Dermatology and Skin Diseases Research Center, University of Alabama at Birmingham, 1670 University Boulvard, VH 566A, P.O. Box 202, Birmingham, AL 35294-0009, USA; ^2^Veteran's Affairs Medical Center, University of Alabama at Birmingham, Birmingham, AL 35294, USA; ^3^Comprehensive Cancer Center, University of Alabama at Birmingham, Birmingham, AL 35294-0019, USA

## Abstract

Breast cancer remains a major cause of death in women in the developed world. As Toll-like receptors (TLRs) are widely expressed on tumor cells and play important roles in the initiation and progression of cancer, they may thus serve as important targets and have an effective perspective on breast cancer treatment. Expression of TLRs on breast cancer cells and mononuclear inflammatory cells can promote inflammation and cell survival in the tumor microenvironment. Inflammation and cancer are related. It is well known that persistent inflammatory conditions can induce cancer formation, due to production of cytokines and chemokines, which play a crucial role in promoting angiogenesis, metastasis, and subversion of adaptive immunity. TLR signaling in tumor cells can mediate tumor cell immune escape and tumor progression, and it is regarded as one of the mechanisms for chronic inflammation in tumorigenesis and progression. This paper delineates the expression of various TLRs in promotion of inflammation and development of mammary tumors. Understanding the mechanisms through which TLRs on breast cancer cells and inflammatory cells regulate growth, survival, and metastatic progression can make them potential targets for breast cancer therapy.

## 1. Introduction

Breast cancer is the most common cancer among American women, except for skin cancers. The chance of developing invasive breast cancer at some time in a woman's life is a little less than 12%. It is the second leading cause of cancer death in women, exceeded only by lung cancer. The chance that breast cancer will be responsible for a woman's death is about 3% [1]. Although clinical signs of disseminated disease occur in fewer than 10% of women at the time of diagnosis, the disease relapses in the form of metastasis within 5 years of surgery in about half of apparently localized tumors. It is difficult to predict the occurrence of distant metastases since breast cancer is a heterogeneous disease encompassing complex pathologic entities [2]. Thus, there is a need for new and effective breast cancer therapies. 

A dynamic interaction between tumors and the immune system is essential for tumor survival, growth, and metastasis [3]. Tumors are infiltrated with large number of immune cells that constitute a major cell population in the tumor microenvironment. Tumor cells depend on their microenvironment to provide signals for growth, anti-apoptosis, angiogenesis, and metastasis [4]. However, tumor cells are also under the surveillance due to their recognition by immune cells as foreign. Therefore, tumors have to overcome such immune surveillance to progress. Analysis of the interactions between tumor cells and the host's immune system has led to the realization that tumor cells have devised multiple strategies to evade immune attack. Development of an invasive cancer, however, is not only a result of the genetic changes in the tumor cell but also the result of genetic and epigenetic changes within the host. Host cells, including inflammatory cells, endothelial cells, and fibroblasts, are recruited and activated in the microenvironment of transformed cells. The acute inflammatory response might succeed in eliminating the malignant cells, but if not, a chronic inflammatory process develops in conjunction with the dying tumor cells. The subsequent reciprocal interactions between these responding normal host cells and genetically altered cells result in the development of an invasive cancer. There is a constant interplay between the innate and adaptive immune systems, which leads to a protective immune response against pathogens and transformed cells and contributes effectively to discrimination between self and nonself. Persistent protumor immune responses (inflammation), now generally accepted as initiating primary tumor development, are also being recognized as mediators of cancer metastasis. Thus, novel anticancer therapeutic strategies targeting molecular and/or cellular mechanisms regulating these collaborative interactions may provide efficacious relief for metastatic disease [5].

Both infection and sterile tissue injury generate strong immune responses. This paradox was first resolved by Matzinger in 1994, who proposed that our immune system is designed to combat danger, rather than mediate recognition of nonself over self [6]. Pathogen-associated molecular patterns (PAMPs) and endogenous molecules created upon tissue injury, since called damage-associated molecular patterns (DAMPs), signal the threat of either infection or injury to the organism, independently of their nonself- or self-identity [7–10]. Damage-associated molecular patterns (DAMPs) include endogenous intracellular molecules released by activated or necrotic cells and extracellular matrix (ECM) molecules that are upregulated upon injury or degraded following tissue damage. Among the cellular receptors that sense these danger signals, Toll-like receptors (TLRs) represent a key molecular link between tissue injury, infection, and inflammation. TLRs are critical in bridging innate and adaptive immune responses and play a significant role in cancer immunosurveillance [5]. Innate immune cells including natural killer (NK), natural killer T (NKT), and *γδ*T cells play a critical role in protecting the host against cancer [5]. Macrophages and dendritic cells (DCs), in particular, function as major sensors of invading pathogens and transformed cells via the TLRs. Adaptive immunity is crucial to the elimination of pathogens and tumor cells in the late phase of host defense responses and generates more specific tumor immunity and immunological memory [11]. TLRs are known to regulate cancer immunity and tolerance by controlling the suppressive function of regulatory T (Treg) cell and through innate immune responses mediated by other immune cells [11–13]. TLR signaling, critical for innate and adaptive immune responses, has been thought to be restricted to immune cells [14]. However, many studies suggest that tumor cells bear TLRs and that TLR signaling promotes tumor growth and immune evasion [15–17]. TLR activation by DAMPs may initiate positive feedback loops where increasing tissue damage enhances proinflammatory responses leading to chronic inflammation. As TLRs are widely expressed on tumor cells and immune cells and play important roles in the initiation and progression of cancer, they may thus serve as an important target and have an effective perspective on breast cancer treatment. 

Currently, 13 mammalian-TLR analogs have been identified. TLRs 1, 2, 4, 5, and 6 are expressed on the cell surface; TLRs 3, 7, 8, and 9 are found almost exclusively within endosomes. Different TLRs exhibit specificity for pathogen-derived ligands; TLRs 2, 3, 4, 5, 7, and 9 recognize bacterial lipoproteins, double-stranded RNA/poly (I : C), lipopolysaccharides (LPS), flagellin, single-stranded RNA, and CpG-containing DNA, respectively [18–23]. The ligands for TLRs 10, 12, and 13 remain unidentified. TLR10 is expressed in humans but not in mice, TLR8 is not functional in mice and TLRs 11, 12, and 13 are expressed in mice but not in humans. 

There are several studies which suggest that DAMP-mediated inflammation plays a vital role. Necrotic cells were found to induce proinflammatory and tissue repair gene synthesis and cause DC maturation in a TLR2-dependent manner, as a result of the release of their intracellular contents. Other intracellular molecules such as heat shock proteins including HSP70, Gp96, HSP22, and HSP72 and high-mobility group box-1 protein (HMGB1) as well as ECM molecules such as biglycan, tenascin-C, versican, and fragments of ECM molecules including oligosaccharides of hyaluronic acid (HA) and heparan sulfate (HS) have been shown to activate TLRs. TLR1, along with TLR2, was found to be important for the activation of professional antigen-presenting cells by *β*-defensin-3, a host-derived antimicrobial peptide. Self-nucleic acids have also been described as endogenous danger signals, namely, mRNA recognized by TLR3, single-stranded RNA (ssRNA) sensed by TLR7 and 8, and IgG-chromatin complexes recognized by TLR9. TLR2, 4, 7, and 8 were shown to be activated by antiphospholipid antibodies (APL) isolated from patients with APL syndrome [24]. 

The signaling pathways utilized by various TLRs differ, which results in varied cellular responses. For example, TLR3, the receptor for double-stranded RNA couples to the adaptor protein TRIF. In contrast, other TLRs couple to the adapter myeloid differentiation primary response gene 88 (MyD88) [25, 26]. The MyD88-adapter protein recruits IRAKs and TRAF6. The TRAF6 in turn activates TAK1 that phosphorylates and activates the IKK complex resulting in the release and translocation of NF-*κ*B to the nucleus. TAK1 also activates stress-activated protein kinase (SAPK) pathways and activates c-Jun-NH2-kinases (JNK) and p38. The MyD88-coupled TLRs induce the synthesis of cytokines such as TNF-*α*, IL-6, and IL-1, key mediators of the inflammatory response [27, 28]. TLR4, the receptor for LPS, is unique in that it activates both MyD88-dependent and TRIF-dependent pathways [28].

## 2. Inflammation and Cancer Metastasis

The link between inflammation and cancer is well documented [29, 30]. Several inflammatory diseases, including inflammatory bowel disease, increase the risk of cancer. Conversely, in tumors that are epidemiologically unrelated to overt inflammatory conditions (such as breast cancer), the activation of oncogenes can trigger the production of inflammatory molecules and the recruitment of inflammatory cells. In the tumor microenvironment, inflammatory cells and molecules influence almost every aspect of cancer progress, including the metastatic ability of tumor cells [29]. There is biological heterogeneity among tumors with regard to cellular infiltrates, identifying subsets of mononuclear inflammatory cells both at the tumor centre and at the invasive front, which seem to be associated with the occurrence of distant metastasis. Intratumour leucocytes from peripheral blood penetrate the tumor architecture after their phenotypic modification, from the invasive front to the tumor centre. This seems to be a dynamic process in which inflammatory cells and immunomodulatory mediators present in the tumor microenvironment polarize the host immune response towards specific phenotypes impacting on tumor progression [31]. Previously, there were six recognized hallmarks of cancer, namely, unlimited replicative potential, self-sufficiency in growth signals, insensitivity to growth inhibitors, evasion of programmed cell death, ability to develop blood vessels, and tissue invasion and metastasis [4]. Cancer-related inflammation has now emerged as the seventh hallmark of cancer. A group of cytokine proteins, including IL-1, IL-6, TNF-*α*, and RANKL, activate inflammation and are known to augment tumor cells' ability to metastasize by affecting several steps in the cells' dissemination and implantation at secondary sites [29, 32, 33]. Inflammatory cytokines lie downstream of the “master” gene transcription factor NF-*κ*B, for promoting inflammation which is itself activated by them [29]. There is strong evidence that the tumor microenvironment is inflammatory and that activation of the innate immune system plays a role in the progression of cancer [34, 35]. A major source of inflammatory cytokines in the tumor microenvironment, are specialized white blood cells called macrophages. Tumor-associated macrophages assist the malignant behaviour of tumor cells, not only by producing cytokines, but also by secreting growth factors and matrix-degrading enzymes [36–38]. It has long been suggested that there may be common pathways of inflammation shared by responses to infection and to malignancy. Recent evidence indicates that TLRs on macrophages may be critical elements in these common pathways. MyD88 has been reported to activate not only AP-1 and NF-*κ*B subunit p65 and p50, but also c-Rel, C/EBP*β*, and C/EBP*δ*. In case of LPS signaling through TLR4, where NF-*κ*B and AP-1 activities are relatively preserved in MyD88-deficient macrophages, the specific defect in c-Rel and the profound defect in C/EBP*β*/*δ* activation likely accounts for the reduction of IL-12 p40, IL-6, and TNF*α*. The absence of both C/EBP*β*/*δ* specifically in TLR signaling impairs key proinflammatory cytokines without affecting other NF-*κ*B-dependent genes such as I*κ*B*α* [39].

## 3. Toll-Like Receptors in Inflammation-Induced Breast Cancer

Toll-like receptors are expressed on cells of the immune system but there is growing evidence that TLRs are also expressed on tumor cells, where they may influence tumor growth and host immune responses [15]. Activation of TLRs expressed on tumor cells may have profound consequences for tumor growth by factors released after TLR activation. Tumor immune evasion may be facilitated by inhibitory cytokines, inflammatory factors, proteinases, and other small molecules such as nitric oxide [40]. Recent evidence suggests that TLRs also contribute to tumor-cell resistance to apoptosis and increased invasiveness. The human breast cancer cell line MDA-MB-231 was found to express TLR1-TLR10 at both the mRNA and protein levels. TLR4 was found to be the highest expressed TLR in MDA-MB-231. Knockdown of *TLR4* gene in MDA-MB-231 resulted in a dramatic reduction of breast cancer cell viability and inhibition of IL-6 and IL-8 cytokines compared with vector control [41]. Another study highlights the role of TLR9 in highly invasive MDA-MB-231 breast cancer cell line which when activated promotes MDA-MB-231 cell invasion by increasing the activity of matrix metalloproteinase 13 (MMP13), but not MMP8 [42]. Samples of mammary carcinomas with recurrence have also exhibited a significant increase in the mRNA levels of TLR3, TLR4, and TLR9. A significant percentage of tumors also showed TLR4 expression by mononuclear inflammatory cells (21.6%) and TLR9 expression by fibroblast-like cells (57.5%). Tumors with high TLR3 expression by tumor cell or with high TLR4 expression by mononuclear inflammatory cells (MICs), but not TLR9 high fibroblast like cells were significantly associated with higher probability of metastasis [2]. This study highlights the importance of the tumor stromal cells in tumor behavior, and how TLR-induced inflammation on inflammatory cells drives metastatic cascade. 

Synthetic TLR9-ligands (CpG-sequence containing oligonucleotides) stimulated TLR9 expressed on cancer cells as well as various normal cells, including mesenchymal stem cells and stimulated their invasion *in vitro*. This invasion was mediated via downregulation of tissue inhibitor of matrix metalloproteinase-3 (TIMP-3) and through matrix metalloproteinase-13 (MMP-13) activation. Expression of TLR9 isoforms A and B have been detected in clinical breast cancer specimens. Expression of TLR9 and its invasive effects on breast cancer cells has been found to be regulated by estrogen receptor-*α* (ER*α*) and sex steroid hormones. TLR9 expression was also found to be affected by commonly used hormonal cancer therapy bicalutamide [43]. 

Activation of TLR signaling on tumor cells by their ligands can also trigger apoptosis and may have therapeutic effects. For example, in a randomized clinical trial for the efficacy of poly (A : U) dsRNA, therapeutic effect was mediated through TLR3 expressed on tumor cells, and could therefore represent an effective targeted treatment in patients with TLR3-positive cancers. The predictive value of TLR3 expression by tumor cells for the efficacy of Poly (A : U) dsRNA was determined in 194 breast cancer patients enrolled in a randomized clinical trial. However, conventional chemotherapy or *in vivo* injection of poly (A : U), alone or in combination, failed to reduce tumor growth unless an immune-chemotherapeutic regimen of vaccination against tumor antigens was included [44]. 

Recently, TLR5 has been found to be highly expressed in breast carcinomas and activation of TLR5-signaling pathway was found to be overly responsive in breast cancer cells by inhibiting cell proliferation and an anchorage-independent growth. In addition, the secretion of soluble factors induced by flagellin, was found to the growth-inhibition of breast cancer cells in an autocrine fashion. This inhibitory activity was further confirmed *in vivo *using mouse xenografts models of human breast cancer cells [45]. Sites of chronic inflammation are often associated with the establishment and growth of various malignancies including breast cancer. Enhanced neutrophilic and granulocytic infiltration in lungs and bone of the proarthritic and arthritic mice and subsequent increase in circulating levels of proinflammatory cytokines, such as macrophage colony stimulating factor (M-CSF), interleukin-17 (IL-17), interleukin-6 (IL-6), vascular endothelial growth factor (VEGF), and tumor necrosis factor-alpha (TNF-alpha) were found to contribute to the increased metastasis. Breast cancer-associated secondary metastasis was found to be significantly increased in pro-arthritic and arthritic conditions. Breast cancer metastasis was found to be significantly reduced by blocking the IL-17 and COX-2 pathway [46]. Inflammatory TLR signaling has also been shown to promote the attraction and generation of Th17 cells induced by tumor cells and tumor-derived fibroblasts. Enhanced migration of Th17 cells to tumor sites was reported to be due to the expression of chemokines and tumor-derived fibroblasts [47]. 

Therapeutic development targeting TLRs is at early clinical stages. There are currently approximately twenty drugs in preclinical development, with a further dozen or so in clinical trials [48]. There are clearly many options for the targeting of TLRs, because the key function of TLRs is to induce cytokines, which are well validated in these diseases and are successfully being targeted in the clinic. TLRs occur early in pathways and so inhibiting them might be more potent than inhibiting their downstream cytokine targets. Different approaches are being taken to target TLRs. Neutralizing antibodies to TLRs are a feasible option, but only for those on the cell surface, such as TLR2, TLR4, and TLR5. Small-molecule antagonists (e.g., eritoran against TLR4 or ODN-based inhibitors of TLR7) might be a better prospect, but it is hard to predict their off-target effects and efficacy. Because there are kinases on the signaling pathways, these might also be sensitive to inhibition. One major concern here, however, is that such inhibitors might block multiple TLRs and therefore give rise to unwanted immunosuppression. Monotherapies against a specific TLR might not have this problem. Studies on knockout mouse indicate that there is less redundancy in TLRs in relation to inflammation. TLR-based adjuvants also have the potential to yield new agents. Imiquimod is already approved for its antiviral effects, whereas MPL is approved as a vaccine adjuvant. In terms of antagonism, effects of TLR inhibitor, eritoran have been found to be significant but somewhat marginal [49]. 

To further develop more effective TLR therapeutic targeting strategy, there are a few more tasks: further identifying and determining the pathogenesis of challenging medical conditions like cancer; analysis of genetic sequence, molecular structure, epigenetic observations, and functional activities on both animal model and human clinical studies; design of clinical study based on study indication, dosing regimens, drug delivery route or format consideration, and pharmacokinetics; timely and objective assessment of adverse events with details. Targeting TLRs will therefore in all likelihood prevent the induction of many immune and inflammatory proteins. The wide tissue distribution of TLRs, however, may make it difficult to determine whether an agonist or an antagonist will be most effective therapeutically.

## 4. Conclusions

Metastasis is regulated not only by intrinsic genetic changes in malignant cells, but also by the microenvironment. Several studies have demonstrated that sites of chronic inflammation are often associated with the establishment and growth of various malignancies. Toll-like receptors (TLRs) have emerged as sensors that can detect a variety of invading pathogens and malignant cells. Since their discovery a decade ago, TLRs have been shown to be critical for efficient innate and adaptive immunity and the framework of TLR-mediated signaling pathway has been explained. However, TLR activation may be a two-edged sword, with both antitumor and pro-tumor consequences. The general expression of functionally active TLRs by tumor cells and inflammatory cells in the stroma by putative endogenous ligands suggests that TLR signaling may be continually activated and may contribute to tumor progression and metastasis. Understanding TLR function in tumor biology may lead to discovery of new therapeutic targets in cancer therapy.
